# Cost Matrix of Molecular Pathology in Glioma—Towards AI-Driven Rational Molecular Testing and Precision Care for the Future

**DOI:** 10.3390/biomedicines10123029

**Published:** 2022-11-24

**Authors:** Sarisha Jagasia, Erdal Tasci, Ying Zhuge, Kevin Camphausen, Andra Valentina Krauze

**Affiliations:** Radiation Oncology Branch, Center for Cancer Research, National Cancer Institute, NIH, 9000 Rockville Pike, Building 10, CRC, Bethesda, MD 20892, USA

**Keywords:** glioma, biomarkers, genomics, prognosis, cost-effectiveness, precision medicine

## Abstract

Gliomas are the most common and aggressive primary brain tumors. Gliomas carry a poor prognosis because of the tumor’s resistance to radiation and chemotherapy leading to nearly universal recurrence. Recent advances in large-scale genomic research have allowed for the development of more targeted therapies to treat glioma. While precision medicine can target specific molecular features in glioma, targeted therapies are often not feasible due to the lack of actionable markers and the high cost of molecular testing. This review summarizes the clinically relevant molecular features in glioma and the current cost of care for glioma patients, focusing on the molecular markers and meaningful clinical features that are linked to clinical outcomes and have a realistic possibility of being measured, which is a promising direction for precision medicine using artificial intelligence approaches.

## 1. Introduction

Gliomas are the most common and aggressive primary brain tumors, with glioblastoma (GBM) resulting in a median survival rate of ~15 months and a 5-year survival rate of 10% [1]. The poor prognosis of glioma and GBM in particular is multifactorial, involving resistance to radiation and chemotherapy, morphological and genetic heterogeneity within individual tumors, tumor alteration over time, and stem cell contribution to resistance, all resulting in nearly universal recurrence [1]. The current standard of care management of glioma involves maximal surgical resection followed by radiotherapy with concurrent and/or sequential and adjuvant temozolomide (TMZ) [1,2]. Radioresistant and chemo-resistant glioma stem cells (GSCs) may in theory be targeted using novel therapies aiming to reduce the risk of tumor recurrence [3,4]. Due to molecular heterogeneity, targeted therapies using these subtypes have not become standard of care due to a lack of precision in patient selection [5]. Given the ability to leverage omic information, a more personalized approach to target specific GSCs might prove to be more effective in the treatment of gliomas. Classification methods employing genomic data extracted from GSCs may also aid in targeting treatment. Previous classification efforts of GBM tumors based on the IDH gene and methylation of the MGMT promoter with respect to clinical value have been described [6]. Recent advances in large-scale genomic research techniques and epigenomic studies involving repositories such as The Cancer Genome Atlas (TCGA), Chinese Glioma Genome Atlas (CGGA), and others could allow for the development of more targeted techniques to treat glioma; however, not all identified genomic signals translate into tumoral or proteomic alterations, and likely only a subset of these connect to clinical tumor behaviors and outcomes secondary to widespread pathway redundancy and secondary heterogeneity that is as yet poorly understood. The high cost of genomic analysis also means that not all patients can benefit from sequencing, diminishing data in this space and limiting future personalized approaches in management. AI may enable the identification of biological patterns in large-scale data to move towards the integration of omic data streams, imaging, and clinical factors via robust, realistically obtainable biomarkers [7,8,9,10,11]. The current review seeks to summarize the significant clinical biomarkers of glioma given that those signals with clinical connections may more realistically translate into clinically actionable biomarkers that can be targeted therapeutically. In Section 2 and Section 3, we will review commonly reported alterations in glioma, exploring current relevant studies. In Section 4 and Section 5, we will report on the cost of care of glioma with reported alterations and omic analysis. Finally, in Section 6, we will present guidelines linking relevant alterations and computational analysis with cost-matrix-guided motivation for artificial intelligence (AI) and rational feature engineering that allows leveraging omics while mitigating health disparities and lack of large-scale data to improve glioma outcomes.

## 2. Molecular Alterations in Glioma

**Figure 1:**
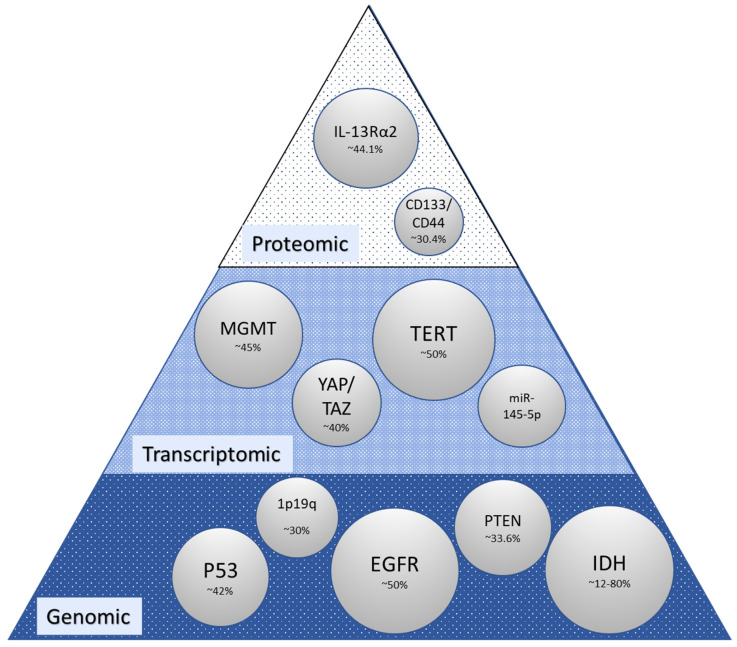
Molecular alterations in glioma [17,18,19,20,21,22,23,24,25,26].

Reported alterations in glioma are broadly categorized as genomic, transcriptomic, and proteomic. Their frequency is highly heterogenous (Figure 1; bubble size indicates alteration frequency reported in glioma in the current literature), and robust connections between these planes are as yet lacking. The most significant points of discussion in this area are molecular subclasses with significant prognostic value (e.g., proneural, neural, classical, and mesenchymal), which were initially identified in 2010 and have been refined since [5,12,13], and markers such as MGMT, IDH, and EGFR alterations that occupy most of the literature in this area (Table 1). Molecular subclasses initially identified four subsets with genomic alterations that correlated to survival. Within this initial classification, proneural and neural patients had the best outcomes, classical had intermediate prognosis, and mesenchymal had the worst outcomes [14]. The proneural subtype has more frequent mutations in PDGFRA (platelet-derived growth factor receptor alpha), the neural subtype has frequent mutations in CD44 and VEGF (vascular endothelial growth factor), classical has frequent EGFR amplification, while the mesenchymal subtype has NFI (neurofibromatosis type 1) and PTEN (phosphatase and tensin homolog) mutations [5,13]. Furthermore, recurrent tumors shift toward the Mes subtype with the worst outcome [5]. Markers such as MGMT (O6-methylguanine-DNA methyltransferase) likely reflect alterations that cross several omic boundaries. MGMT is one of the most widely studied markers in glioblastoma. Some studies show no association between the MGMT promoter and GBM’s molecular markers such as ATRX (ATP-dependent helicase), IDH (isocitrate dehydrogenase), p53, and Ki67 [15]. However, patients with a methylated MGMT promoter benefit from TMZ [2,16] as compared to those without a methylated MGMT promoter. The MGMT promoter is methylated in 45% of GBM and is associated with a prolonged overall progression-free survival [17].

The most clinically significant gene researched to date is the IDH gene which codes for the isocitrate dehydrogenase enzyme [27]. IDH genes are responsible for the intramitochondrial enzyme with three intracellular forms. IDH mutations were found in 12% of GBM patients and 80% of glioma patients in a cohort at Duke University [26]. Patients with the IDH1 gene mutation have been linked to improved intracellular response to TMZ when compared with individuals with the wild-type *IDH1* gene [6]. However, IDH1 mutation status does not predict progression-free survival [28,29]. Codeletion of gene 1p19q is associated with a better response to radiation therapy and chemotherapy and longer progression-free survival. This codeletion is present in around 30–50% of gliomas [22]. The EGFR gene is an oncogene in the RTK signaling pathway that encodes cell surface receptor tyrosine kinase involved in DNA transcription, anti-apoptosis, angiogenesis, and cellular proliferation [30]. EGFR amplification is present in approximately 40–50% of primary GBMs [23]. The EGFRvIII mutation and EGFR amplification in particular have been shown to have contradictory clinical results [12]. YAP and TAZ transcription coactivators are highly expressed in EGFR-amplified/mutant GBM cells. Disrupting YAP/TAZ-mediated transcription can induce apoptosis and reduce proliferation in EGFR-amplified cells [31]. Altered expression of YAP and TAZ occurs in 40% of gliomas [31].

TP53 is a tumor suppressor gene in the p53 signaling pathway [27]. This pathway has been implicated in cell invasion, migration, proliferation, evasion of apoptosis, and cancer cell stemness [32]. TP53 has a mutation frequency of 42% [23]. The p53 signaling pathway is downregulated in 84% of GBM patients [32]. Micro RNA (miRNA) and long non-coding RNA (ncRNA) are also responsible for regulating various pathways in glioma including the p53 pathways responsible for tumor suppression [32]. The expression of various miRNAs and ncRNAs is found to regulate various forms of GBM. Specifically, the miRNA miR-145-5p is a highly reliable diagnostic indicator of GBM [21]. High levels of miR-145-5p expression are linked to longer overall survival times compared to lower levels [21]. MiR-145-5p does not have data alteration frequency yet.

PTEN is a tumor suppressor gene in the PI3K signaling pathway [27]. An analysis of TCGA data identified mutated PTEN in 33% of GBM [23]. PTEN is responsible for regulating glucose metabolism through the P13K-AKT pathway with deletion correlating to poor prognosis in GBM patients [33]. Mutations of the PI3K/mTOR pathway occur in nearly 50% of GBMs [3]. The telomerase reverse transcriptase (TERT) gene promoter mutation was found to be in 80.3% of primary GBM and 28.6% of secondary GBM and found to predict overall poor survival in patients who had incomplete resections and no temozolomide chemotherapy [34]. The TERT promoter mutation in combination with EGFR amplification and IDH mutation status improves the prognostic classification of GBMs [35]. NF1 is a tumor suppressor gene in the MAPK signaling pathways [36]. In mesenchymal tumors, the NF1 protein is downregulated which upregulates the MAPK signaling pathways [6]. The P13K-AKT pathways are frequently deregulated through NF1 and PTEN co-mutation in the mesenchymal subtype [5]. The CD44 genes encode the transmembrane glycoprotein and have been known for regulating tumorigenesis [37]. High expression of CD44 leads to cancer cell proliferation, motility, and survival and promotes cancer metastasis [38]. CD133 is co-expressed with CD44 and linked to similar molecular features [19]. IL-13Ra2 is an interleukin receptor that is overexpressed in over 60% of GBM and 44.1% of gliomas [39]. Clinical trials have found immunotherapies targeting the IL-13Ra2 receptor to be effective [39].

In addition, epigenetic and translational modifications including histone post-translational modifications play a significant role in GBM development and progression [18]. Enzymes such as histone deacetylase and demethylates (HDMTs/KDMTs) have been found to be deregulated in GBM. Of note are the enzymes lysine and arginine methyltransferases (G9a, SUV39H1, and SETDB1), acetyltransferases, and deacetylases (KAT6A, SIRT2, SIRT7, HDAC4, 6, 9) that are dysregulated in GBM but more data are needed before these enzymes become clinically relevant [40]. Based on currently available TCGA and CGGA data, the most frequently reported molecular/genomic features in glioma are IDH, TP53, ATRX, PTEN, EGFR, CIC, MUC16, PI3K3CA, NF1, PIK3R1, FUBP1, TB1, NOTCH1, and TERT (Tasci et al., unpublished data) [41], and given their capture and potential for analysis, these can help make connections to MGMT, IDH, and other markers and clinical outcomes. Still, there is no clear relationship between IDH1, p53, and MGMT alterations, making analysis on glioma difficult. Furthermore, it should be noted that both MGMT and IDH status are often not available for analysis in data sets with greater than 40% of cohorts missing MGMT methylation status [42], and missing data are thus not analyzed nor easily or transparently recognized. Using deep learning methods and radiomic features to predict missing data such as MGMT methylation status has shown promise; however, more research on deep learning models and molecular models is needed before they can be applied in clinical decision making [43]. Previous attempts at glioma classification including by Verhaak et al. [5] did not carry over into the clinic even though they identified important associations, in part due to cost but also due to a lack of clear connection to other markers such as MGMT and IDH which are actively being measured in the clinic and, more significantly, because of a lack of connection to clinical features that define the outcome and clinical decision making in the real world. Another important facet of the discussion of linking clinical outcomes and molecular/genomic features is the question of how AI can learn from existing data of which current analyses struggle to compare different measures, as evidenced by comparing expression data to mutation data to downstream omic data that is dynamically regulated in an area of extreme biological heterogeneity where genomic classification does not yet clearly connect to pathology nor molecular or clinical endpoints [6,16,44]. AI holds the promise of harnessing large-scale data to identify the most relevant signals that define a response or resistance to treatment, features which, once validated, can be carried over into the clinic to direct management to, for example, augment standard of care management with additional molecular targeted agents, alterations in the number of cycles of adjuvant TMZ following concurrent CRT, or biologically optimized radiation therapy dose and dose distribution. The optimization of all aspects of care holds the promise to advance outcomes but also more effectively channels the cost of care. Clinical and molecular features in glioma will be discussed in the next section.

**Table 1:** Relevant studies in Glioma. ** Denotes major literature review on the topic.

Authors	Title	Finding
Kitange et al., 2009 [16]	Induction of MGMT expression is associated with temozolomide resistance in glioblastoma xenografts	MGMT expression is dynamically regulated in some MGMT nonmethylated tumors, and in these tumors protracted dosing regimens may not be effective.
Delfino et al., 2011 [45]	Therapy-, gender- and race-specific microRNA markers, target genes and networks related to glioblastoma recurrence and survival	Sensory perception and G-protein-coupled receptor processes were enriched among microRNA gene targets also associated with survival, and network visualization highlighted their relations.
Håvik et al., 2012 [46]	MGMT promoter methylation in gliomas-assessment by pyrosequencing and quantitative methylation-specific PCR	MGMT promoter methylation analysis gives sufficient prognostic information to merit its inclusion in the standard management of patients with high-grade gliomas, and in this study pyrosequencing seemed the better analytical method.
Aldape et al., 2015 [6] **	Glioblastoma: pathology, molecular mechanisms and markers	IDH-mutant GBMs are clearly distinct from GBMs without IDH1/2 mutation with respect to molecular and clinical features, including prognosis.
Tanguturi et al., 2017 [47]	Characterization of MGMT and EGFR protein expression in glioblastoma and association with survival	There were several associations between GBM genomic subgroups and clinical or molecular prognostic covariates and validated known prognostic factors in all survival periods.
Asif et al., 2019 [27]	Comparative proteogenomic characterization of glioblastoma	Significantly mutated genes in GBM included TP53, EGFR, PIK3R1, PTEN, NF1, RET, and STAG2. MGMT methylation was present in two-thirds of cases.
Burgenske et al., 2019 [9]	Molecular profiling of long-term IDH-wildtype glioblastoma survivors	Unique attributes were observed in regard to altered gene expression and pathway enrichment. These attributes may be valuable prognostic markers and are worth further examination.
Gobin et al., 2019 [48]	A DNA Repair and Cell-Cycle Gene Expression Signature in Primary and Recurrent Glioblastoma: Prognostic Value and Clinical Implications	Classification of GBM tumors based on a DNA repair and cell cycle gene expression signature exposes vulnerabilities in standard-of-care therapies and offers the potential for personalized therapeutic strategies.
Neftel et al., 2019 [36]	An Integrative Model of Cellular States, Plasticity, and Genetics for Glioblastoma	Malignant cells in glioblastoma exist in four main cellular states that recapitulate distinct neural cell types, are influenced by the tumor microenvironment, and exhibit plasticity. The relative frequency of cells in each state varies between glioblastoma samples and is influenced by copy number amplifications of the CDK4, EGFR, and PDGFRA loci and by mutations in the NF1 locus, which each favor a defined state.
Oh et al., 2020 [49]	Integrated pharmaco-proteogenomics defines two subgroups in isocitrate dehydrogenase wild-type glioblastoma with prognostic and therapeutic opportunities	Two distinct binary classifications of IDH wild-type GBM tumors. GBM proteomic cluster 1 (GPC1) tumors exhibit Warburg-like features, neural stem cell markers, immune checkpoint ligands, and a poor prognostic biomarker, FKBP prolyl isomerase 9 (FKBP9). Meanwhile, GPC2 tumors show elevated oxidative phosphorylation-related proteins, differentiated oligodendrocyte and astrocyte markers, and a favorable prognostic biomarker, phosphoglycerate dehydrogenase (PHGDH).
Mata et al., 2020 [50]	Genetic and epigenetic landscape of IDH-wildtype glioblastomas with FGFR3-TACC3 fusions	Despite being older at diagnosis and having similar frequencies of MGMT promoter hypermethylation, patients with F3T3-positive GBMs lived about 8 months longer than those with F3T3 wild-type tumors. Consistent with IDH wild-type GBMs, F3T3-positive GBMs exhibited distinct biological features.
Egaña et al., 2020 [15]	Methylation of MGMT promoter does not predict response to temozolomide in patients with glioblastoma in Donostia Hospital	No association was detected between methylation of MGMT promoter and molecular markers such as ATRX, IDH, p53, and Ki67.
Schaff et al., 2020 [44]	Characterization of MGMT and EGFR protein expression in glioblastoma and association with survival	A weak association was seen between MGMT protein expression and promoter methylation. Quantification of MGMT protein expression was inferior to MGMT methylation for prognostication in GBM.
Cong et al., 2021 [51]	Identification of the Role and Clinical Prognostic Value of Target Genes of m6A RNA Methylation Regulators in Glioma	The study established and validated a seven-gene signature comprising METTL3, COL18A1, NASP, PHLPP2, TIMP1, U2AF2, and VEGFA, with a good capability for predicting glioma survival. These genes were identified to influence 81 anticancer drug responses, which further contributes to the early-phase clinical trials of drug development.
Digregorio et al., 2021 [52]	The expression of B7-H3 isoforms in newly diagnosed glioblastoma and recurrence and their functional role	B7-H3 was a marker for SVZ-GBM cells. B7-H3 inhibition in GBM cells reduced their tumorigenicity. Out of the two B7-H3 isoforms, only 2IgB7-H3 was detected in non-cancerous brain tissue, whereas 4IgB7-H3 was specific to GBM. 2IgB7-H3 expression was higher in GBM recurrences and increased resistance to temozolomide-mediated apoptosis.
Syafruddin et al., 2021 [37]	Integration of RNA-Seq and proteomics data identifies glioblastoma multiforme surfaceome signature	The results identify six high-confidence GBM genes, HLA-DRA, CD44, SLC1A5, EGFR, ITGB2, PTPRJ, which were significantly upregulated in GBM. High expression of CD44, PTPRJ, and HLA-DRA was significantly associated with poor disease-free survival.
Wang et al., 2022 [53]	Identification of Prognostic Biomarkers for Glioblastoma Based on Transcriptome and Proteome Association Analysis	Fibronectin 1(FN1) was a prognostic risk factor and significantly upregulated in GBM samples. FN1 may play a role in GBM progression through ECM-receptor interaction and PI3K-Akt signaling pathways.

## 3. Clinical and Management Features of Significance in Glioma

**Figure 2:**
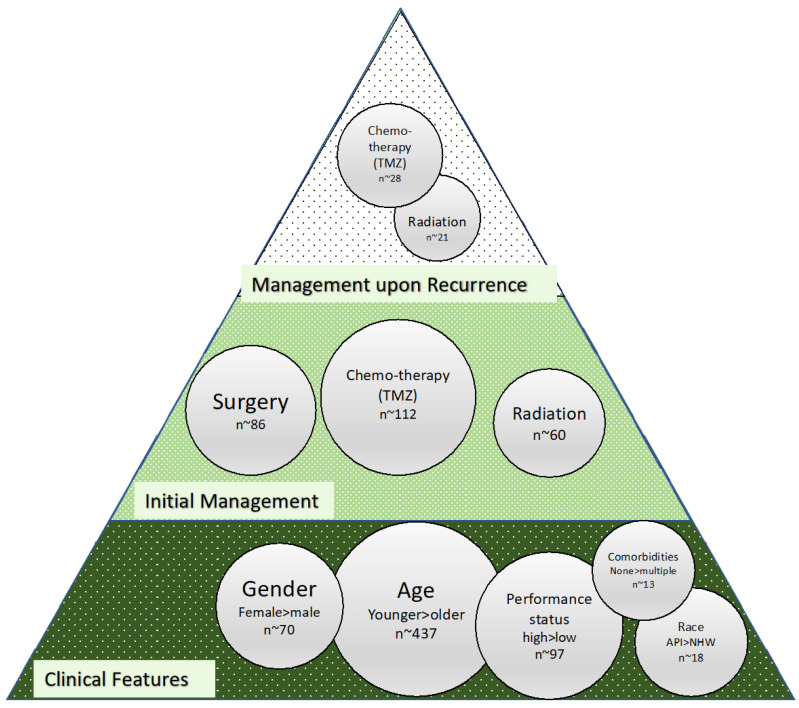
The current literature on patient characteristics is reported to be associated with outcomes in glioma (n indicates the number of articles reported in a PubMed search from 2012 to 2022) [73]. Bubble size for clinical features and treatments was determined based on PubMed search findings. MeSH terms used in the search for clinical features were “glioma”, “prognosis”, and “indicates” and for clinical features either “race”, “sex”, “performance status”, “age”, or “sex.” Terms used for initial management therapies were “glioma”, “prognosis”, “indicates”, “initial”, and either “surgery”, “radiation”, or “chemotherapy”. For recurrent tumor treatment bubbles, the same search was used with the term “recurrent” instead of “initial.” All searches were filtered for only articles in the last 10 years.

Clinical and disease features are most often employed as a means of elucidating prognosis in glioma. The patient-related factors most frequently associated with prognosis are age, performance status, gender, race, and comorbidities (Figure 2). The relationship between age and prognosis has benefited from several analyses including a recent analysis of the SEER database from 2000 to 2018 wherein a nonlinear relationship was revealed with the hazard ratio of death increasing to 10 years, then decreasing to 23 years, and subsequently becoming J-shaped with increasing age [54] in contrast to previously described groupings based on smaller groups of patients, where the age-prognosis relationship was described more rigidly along age lines [55], and older studies where age was dichotomized to less than or greater than 50 years old such as in the context of traditional recursive partitioning analysis (RPA) [56]. There is also reported intersectionality between age and gender and between these clinical features and molecular markers with GFAP, EMA, MGMT, P53, NeuN, Oligo2, EGFR, VEGF, IDH1, Ki-67, PR, CD3, H3K27M, TS, and 1p/19q status included in the age group classification by Lin et al. 2020 [55]. Gender-related prognosis differences and associated molecular features support connections to outcomes [57,58]. Molecular differences associated with gender were reported for XIST, PUDP, ZFX, JPX, KDM6A, and TSIX in females and PRKY, RPS4Y2, PCDH11Y, EIF1AY, RPS4Y1, and ZFY in males when the analysis was carried out on GBM and LGG [58]. With respect to race and ethnicity, significant intersectionality exists between the risk of glioma, health disparities, and genetic heterogeneity, all of which impact prognosis [59], with recent data showing similar mortality risks for black, Hispanic, and white patients and superior survival reported in non-Hispanic Asian/Pacific Islander patients [60], and SEER data from 2002 to 2014 showing non-Hispanic white patients having higher incidence and lower survival as compared to other ethnic groups [61]. An association between genetic pathways underlying glioma with white patients having a higher risk for glioma than non-white patients and gliomas from white patients less likely to have p53 mutation was reported in 2001 [62]. Increasingly, data are becoming available in which molecular features with links to race have been identified, including with respect to TP53 and EGFR [63]. However, a clear relationship between molecular alterations and ethnicity/race is lacking. Performance status has been extensively reported on in glioma patients as a prognostic marker [64,65,66]. It is a component of RPA but is also often poorly captured and less likely to have an immediate connection to molecular features even as it may well connect to comorbidities, another clinical facet that is poorly captured but may have a molecular signature. Additional clinical features related to the upfront management of glioma begin with the extent of resection [67]; administration of chemotherapy, in particular the extent of adjuvant temozolomide in terms of the number of cycles; and additional management upon recurrence (Figure 2) for which molecular markers are not specifically identified. Figure 2 indicates clinical features with significant prognostic value for patients with glioma and current management protocols.

Metabolic markers can provide additional information on GBM’s response to therapies and progression. Previous research has found a significant correlation between mutations found in GBM and the tumor’s metabolic fingerprint [68]. Metabolic markers in conjunction with genomic, radiomic, and proteomic data should be used to develop clinical models. To overcome the heterogeneity of glioma, several AI algorithm application tools have been used. In particular, combining imaging techniques (i.e., CT, MRI, PET-CT) with metabolic markers and proteome data has been shown to yield useful information for clinical applications [69]. Current models predict overall survival, progression-free survival, and molecular subtypes of high-grade glioma as well as genetic alterations. Models using radiomic features have been shown to outperform models using clinical data—particularly patient age, the Karnofsky performance scale, surgical resection, and genetic alterations—in GBM outcome prediction [70]. Using ensemble models, models using more than one machine learning algorithm, as opposed to models using only one machine learning algorithm, can help overcome the lack of standardization of radiomic features. Pasquini et al. showed that certain radiomic features can be used to predict molecular markers, indicating a correlation between imaging data and tumor histology [71]. Previous prognostic models using stemness-based classification can be used to guide treatment decisions in selecting potential responders for preferential use of immunotherapy [72].

The thought process embedded in clinical decision making is ultimately a byproduct of the reported data (Figure 2) and available markers in the clinic (MGMT, IDH, and others), these linking to the overall cost of care which translates into the type of data that is ultimately available for AI-driven approaches and which will be discussed next.

## 4. The Cost of Care and Omics in Glioma

The cost of care and management of a patient with glioma is comprised of the sum of the costs of imaging, laboratory work, surgical resection, pathology analysis, sequencing when available, radiation therapy (RT), concurrent temozolomide (TMZ), adjuvant TMZ, additional systemic treatments including novel interventions, lost wages for sick patients, and additional surgical intervention and/or additional RT as well as the cost of supportive care. Cost-effectiveness studies in glioma have previously examined several aspects of glioma care [74], including notably surgical intervention [75], chemotherapy and radiation [76], and novel interventions including imaging [77,78]. Molecular pathology has emerged as perhaps the single most important feature of discussion in tumor boards given both its relationship to outcomes and the potential for its leverage in altering management. As a result, molecular pathology was responsible for shifting glioma staging from morphological-based grading to molecular classification in the 2016 iteration of CNS classification [79]. However, even as molecular features gained importance, they also became a divisive issue of accessibility with rising health care expenses and molecular-based access to treatment options. This has prompted justified debate on its bioethical implications and contribution to health disparities already at play in the field [80]. The NHS national costing project carried out a cost-effectiveness study employing a patient cohort originating in a neuro-oncology clinic at a university teaching hospital, including 236 patients treated between 1989 and 1995. In this period, they identified a total cost of GBP 1978 to GBP 26,980, with neuropathology (GBP 434) and chemotherapy (GBP 440) representing a relatively smaller proportion of the cost and radiotherapy (GBP 8832) the largest [74]. In more recent studies, however, authors note the difficulties in carrying out cost analyses given the lack of QALY as the outcome measure and the prevalent use of overall survival or life years gained (LYG) [75], as well as the lack of quality of life values for specific health statuses or utilities [75]. A more recent study examining the cost of care in 13 countries in a global context confirms these limitations in determining the cost of care in glioma [81]. A retrospective claims database analysis from 2019 also notes this but provides more distinct numbers, reporting mean total per patient costs at 6 and 12 months of USD 117,325 and USD 162,550 for first-line treatment and USD 126,128 and USD 243,833 for the second line, with costs driven by the cost of RT and systemic cancer therapy [82]. Data are also emerging for novel interventions, as exemplified in a 2019 study by Butenschon et al. in which the incremental cost-effectiveness ratios varied from EUR 8325 per QALY (5-ALA) to EUR 518,342 per LYG (tumor treating fields) [75]. Some interventions can have their cost-effectiveness examined in relation to specific sources of data, as is the case in a recent study looking at the continuation of adjuvant temozolomide using novel imaging to assess response. 18F-FET PET was found to increase the rate of correctly identified responders to chemotherapy by 26%, with four patients needing an 18F-FET PET to identify one additional responder, and, when compared to MRI, the ICER resulted in EUR 4396.83 for each additional correctly identified responder using 18F-FET PET [77]. The specific potential costs of neuropathology will be discussed next.

## 5. The Cost of Neuropathology and Omics in Glioma

**Figure 3:**
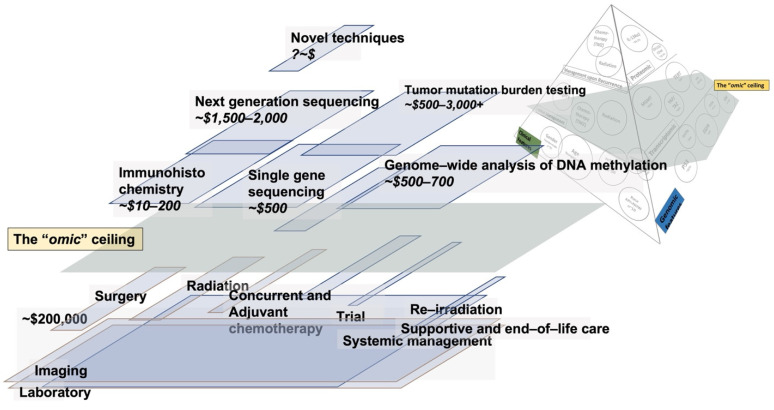
The cost of care and omics in glioma and emergence of the omic ceiling [74,75,76,78,82,84,85,86,89,90,91,92].

Given more widely available data in clinical management, siloed, variable, and fragmented molecular data results in an “omic” ceiling (Figure 3), the extent and placement of which may vary based on management setting, geographical location, and socioeconomic factors that link cost and reimbursement. MGMT is prognostic and predictive but has been challenging to implement across the board, in part due to cost. There are several MGMT detection methods and hence variability in its capture, and the results also produce varying cost parameters [83]. Cost-effectiveness testing for 1p19q [84] and IDH [85] has recently been carried out. Concerning IDH mutation testing, a 2017 study reported on 1023 IDH tests carried out between 2010 and 2015, costing USD ~1.09 million in direct laboratory test costs [85]. The authors proposed an age cutoff of 55 years for glioblastoma patients, which would result in savings of USD 403,200, and not performing sequencing in patients ≥55 years, which would reduce turnaround times by 53%, allowing patients to benefit from an earlier diagnosis [85]. The authors reported on a cost model generated using costs of USD 135 per p.R132H-specific IDH1 immunohistochemistry, USD 420 for single gene sequencing and USD 1800 for next-generation sequencing, and turnaround times of 2 days for immunohistochemistry and 14 days for next-generation sequencing. With respect to 1p19q, a 2022 Cochrane review found no relevant economic evaluations [84]. FISH (fluorescence in situ hybridization) was employed as the reference standard, and MLPA (multiplex ligation-dependent probe amplification) and CISH (chromogenic in situ hybridization) were both found to be cost-effective, with the nuance that the cost-effectiveness of the test was driven by the value placed on detecting a true positive (if society was willing to pay GBP 1000 or less for a true positive detected) and contingent on the outcome measure (a true negative detected or correct diagnosis). The authors noted that as threshold values increased, none of the tests were more likely to be considered cost-effective [84]. With respect to molecular information, NGS (next-generation sequencing) represents the single most significant cost, with attempts discussed here to mitigate the costs by restricting its use to certain age groups, which, as indicated by DeWitt et al., would result in USD ~23.7 million of laboratory cost savings in the IDH testing scenario [85]. A real-world examination of the molecular testing trends in NGS testing in the US revealed significant variation and utilization, with a cost of USD 1269 to USD 2058 per NGS test and tumor mutation burden testing (TMB) ranging from USD 438 to USD 3700 per test [86], with a trend towards a decrease in cost over time. However, given the high cost of NGS and competing pressures on health care systems, including secondary to the COVID-19 pandemic, reimbursement of NGS is lacking, and as a result its implementation into clinical care continues to foster health disparities. In a 2020 study examining reimbursement for genomic panels, the authors found that 77% of tests were denied coverage, with insurers reimbursing 10.75% of the total NGS service charge [87]. The other dimension of the cost discussion relates as much to the cost itself as it does to the ability to direct the cost to clinically meaningful outcome improvements, as noted earlier with respect to imaging directing, stopping, or continuing systemic management or, even more significantly, the selection of alterations that carry clinical impact. When not covered, costly sequencing costs may be absorbed by the patient resulting in significant hardship, which is particularly unjustified when the alterations do not carry sufficient weight to direct care or improve outcomes. Given the cost of NGS and the small proportion of alterations identified that currently visibly direct care, the cost per clinically impactful alteration is exceedingly high. Nonetheless, when NGS can help direct care by improving pathological diagnosis, management, and survival, the cost may be justified [88].

## 6. Optimizing Cost-Benefit in Glioma to Advance Patient Outcomes

The cost/benefit optimization rationale requires scrutiny of all aspects of the process to allow for broader implementation in the clinic in a manner that improves patient outcomes and decision making but, most importantly, does so while accounting for data that are both realistically generated and used for decision making in the clinic (Figure 3). Thus, the molecular “cost” begins with sample acquisition, a practical conundrum for patients and providers as well as a serious cost consideration. NGS requires tissue which arguably is harder to reacquire upon recurrence given the limitations due to patient performance status, tumor location, and comorbidities in the context of tumor molecular evolution over time. It is also challenging to distribute and/or harness limited tissue for multiple analyses and for sharing with other institutions, as is the case with tumor blocks and tissue slides, and which is subject to sampling limitations. This limits the ability to examine change over time, which would allow for AI approaches to learn from changing mutational and biological patterns. NGS is also more costly compared to other tests. Thus, while NGS may represent a feasible cost-effective baseline option for upfront patient selection for the most appropriate treatment, additional and alternative avenues will realistically be needed to determine which patients’ management was effective and which patients require a change in management to merge optimal management with tumor alterations over time. In this context, serum/plasma are more easily acquired and offer an avenue to resample which can capture tumor evolution over time, with less concern for the sampling of the tissue itself and the ability to measure biomarkers in real time [93]. This can allow for the analysis of circulating tumor cells, mRNA, DNA, and exosomes. Analysis of the wide-ranging data that can emerge from this avenue is, however, lacking and carries significant dimensionality, although its relevance to actionable biomarkers is augmented by other data including imaging [94]. Previous research has used transcriptomic data to develop prognostic models for GBM patients [75]. Machine learning prognostic models using a combination of imaging patterns, clinical features, molecular markers, and radiomic data have shown promising results [69,95,96]. The rational implementation of AI algorithms—especially in areas such as imaging where data are already generated as part of the standard of care, as is the case with MRI of the brain for glioma patients—are crucial, since this forms the “floor” or base from which diagnosis and management originate in glioma and are already built into the cost of care (Figure 3). Considering the biological heterogeneity of glioma, imaging techniques are increasingly being harnessed to augment both clinical and molecular data towards precision care and perform exceedingly well, including outperforming more traditional models that harness clinical information and genetic alteration [71,72]. Imaging offers the unique opportunity to realistically and quantitatively link real-time tumor evolution to molecular and genetic alterations [71]. The data themselves, in terms of the type of imaging sequences acquired, their standardization, and the most optimal analysis while employing AI are evolving rapidly, with benchmarks moving towards assessing the most effective set of algorithms to optimize predictions given the data-subtype-specific needs to achieve transferability [70,71]. To optimize cost-benefit, feature selection will need to be benchmarked across data sets to identify the most important feature subsets from all available features. Feature selection aims to remove unrelated, insignificant, and redundant predictors by improving the learning model performance and/or reducing the computational cost, thus increasing efficiency [41,94,97]. In general, feature selection methods are categorized into three categories: filter, wrapper, and embedded methods. Additional information, methods, and case studies for oncologic data can be obtained from [94]. The selection of the best subset of molecular and clinical features by reducing the number of features with various feature selection methods is crucial to allow for cost-effective glioma grading and improvement in patient outcomes. Given the widely studied molecular markers (Section 2) and meaningful clinical features (Section 3), those markers that link to clinical outcomes and have a realistic possibility of being measured in the real world with reliable data capture will result in the most optimal path to precision medicine in glioma, given the attention to optimal feature engineering and mitigation of bias [94].

## 7. Future Directions

Currently, IDH and MGMT methylation are the only two molecular features more widely implemented in treating glioma patients. Other significant molecular features include PTEN, EGFR, 1p19q, p53, NF1, YAP/TAZ, TERT, miR-145-5p, IL-13Ra2, and CD133/CD44. The availability of molecular testing in glioma and its integration in the development of treatments means treatment selection can be guided by MGMT methylation status, IDH1 gene mutation, codeletion of 1p19q, and p53 status. Given the high cost of care for glioma patients and the value of molecular testing in treating glioma, identifying and growing data of clinically relevant molecular features to break the omic ceiling will allow for cost-effective glioma classification and, in doing so, improved patient outcomes. However, before breaking the “omic” ceiling, AI can help maximize the use of the data emerging from the “floor” or base of the cost of care pyramid through feature selection and optimal predictive AI algorithms. Developing new models that leverage clinical, imaging, and molecular features using AI, while considering the cost of care in glioma and emergent data would drive motivation towards clinically explainable and trustworthy clinical results that employ large-scale data and would allow targeted therapies to become more feasible as they are being developed. More research is needed to identify the molecular features with the most clinical value to achieve this as we look to diminish omic health disparities to pave the way for clinically meaningful data allowing AI-driven precision care to best serve patients with glioma.

## Abbreviations

CD44	Cluster of Differentiation 44
CD133	Cluster of Differentiation 133
CGGA	Chinese Glioma Genome Atlas
CISH	Chromogenic In Situ Hybridization
CNS	Central Nervous System
EGFR	Epidermal Growth Factor Receptor
FISH	Fluorescence In Situ Hybridization
GBM	Glioblastoma
GSC	Glioma Stem Cells
HDMT	Histone Deacetylase
ICER	Incremental Cost-Effectiveness Ratio
IDH	Isocitrate Dehydrogenase
KDMT	Histone Demethylates
LGG	Low-Grade Glioma
LYG	Life Years Gained
MAPK	Mitogen Activated Protein Kinases
MGMT	O6-methylguanine-DNA Methyltransferase
MLPA	Multiplex Ligation-Dependent Probe Amplification
MRI	Magnetic Resonance Imaging
NF1	Neurofibromatosis 1
NGS	Next-Generation Sequencing
NHS	National Health Service
PDFRA	Platelet-Derived Growth Factors
PTEN	Phosphatase and Tensin Homolog
P13K-AKT	Phosphoinositide 3-kinasem-Protein Kinase B
QALY	Quality-Adjusted Life Years 18-FET-PET
RPA	Recursive Partitioning Analysis
RT	Radiation therapy
SEER	Surveillance, Epidemiology, and End Results
TAZ	WW Domain-Containing Transcriptional Regulator 1
TCGA	The Cancer Genome Atlas
TERT	Telomerase Reverse Transcriptase
TMB	Tumor Mutation Burden Testing
TMZ	Temozolomide
TP53	Tumor Protein p53
YAP	Yes-Associated Protein
VEGF	Vascular Endothelial Growth Factor
18F-FET PET	O-(2-[18F]fluoroethyl)-L-tyrosine Positron Emission Tomography
